# B Cell Intrinsic Mechanisms Constraining IgE Memory

**DOI:** 10.3389/fimmu.2017.01277

**Published:** 2017-11-13

**Authors:** Brice Laffleur, Orianne Debeaupuis, Zeinab Dalloul, Michel Cogné

**Affiliations:** ^1^Department of Microbiology and Immunology, College of Physicians and Surgeons, Columbia University, New York, NY, United States; ^2^Faculté de Pharmacie, Université Paris-Descartes, Paris, France; ^3^UMR 7276 Centre National de la Recherche Scientifique: Contrôle de la Réponse Immune B et des Lymphoproliférations, Université de Limoges, Limoges, France; ^4^Institut Universitaire de France, Paris, France

**Keywords:** IgE, B cell receptor, B-lymphocytes, IgE regulation, IgE memory

## Abstract

Memory B cells and long-lived plasma cells are key elements of adaptive humoral immunity. Regardless of the immunoglobulin class produced, these cells can ensure long-lasting protection but also long-lasting immunopathology, thus requiring tight regulation of their generation and survival. Among all antibody classes, this is especially true for IgE, which stands as the most potent, and can trigger dramatic inflammatory reactions even when present in minute amounts. IgE responses and memory crucially protect against parasites and toxic components of venoms, conferring selective advantages and explaining their conservation in all mammalian species despite a parallel broad spectrum of IgE-mediated immunopathology. Long-term memory of sensitization and anaphylactic responses to allergens constitute the dark side of IgE responses, which can trigger multiple acute or chronic pathologic manifestations, some punctuated with life-threatening events. This Janus face of the IgE response and memory, both necessary and potentially dangerous, thus obviously deserves the most elaborated self-control schemes.

## Introduction

B cells are specialized in immunoglobulin (Ig) selection and production, the development of which implies several phases. The first is antigen (Ag) independent, with V(D)J assembly creating functional B cell receptor (BCR) genes. In a second phase after cells have reached the periphery, Ag activation can induce expression of activation-induced deaminase (AID) (1, 2), then allowing a second round of Ig gene diversification by somatic hypermutation (SHM) and/or class switch recombination (CSR), which strongly influences their fate (3). In parallel, activated lymphocytes can further differentiate into memory B cells or into plasma cells (PCs) secreting antibodies. PCs can themselves be split into either short- or long-lived cells, the latter surviving and secreting Ig for years in human. CSR switches Ig production from IgM (^+/−^IgD) to IgG, IgA, and IgE. IgE was the last discovered Ig class (4, 5), notably due to its low amount in body fluids and the scarcity of IgE^+^ cells *in vivo*.

Both IgE and IgG emerged in proto-mammals from the ancestral reptilian IgY molecule and further diverged (6). IgG and IgE now stand as the predominant vs. less abundant Ig class in mammals. IgE structure (i.e., constant domains, positions of disulfide bonds, and a CH3 domain N-glycosylation site) is well conserved, with homology culminating at the Fc receptor high-affinity (FcεRI) binding site (7), in agreement with its major functional role. Tiny production is another IgE conserved feature, indicative of a stringently controlled process, consistent with IgE potentially hazardous properties during anaphylactic reactions (8).

IgE antibodies are notably active against helminths, in responses featuring high total and specific IgE levels, hyper-eosinophilia and Th2 polarization (9). IgE have crucial FcεRI-dependent anti-venom properties (10, 11). They also show an adjuvant effect for IgG responses, dependent on the low-affinity FcεRII receptor (CD23) (12–14), which could participate in Ag endocytosis and presentation to T cells. IgE can finally act against tumors by recruiting eosinophils, mastocytes, macrophages, and CD8 T-cells, in a FcεRI-dependent manner (15–17).

Unfortunately, these benefits come with multiple deleterious effects in allergies, and even also autoimmunity (18, 19). Accordingly, the immune system evolved with multiple regulations tempering IgE responses. IgE CSR is stimulated by Th2 cytokines IL4 and IL13, and by IL9 producing T-cells and mast cells [for review (20, 21)], whereas other cytokines (IFNγ, TGFβ, IL10, etc.), T_REG_ cells, and dendritic cells dampen IgE responses (22, 23). These extrinsic controls are consolidated by B cell intrinsic brakes ensuring tight regulation of IgE CSR, mostly short-lived IgE PC differentiation and an extremely short-lived fate of lymphocytes expressing membrane IgE (mIgE). We review recent findings documenting how IgE production is intrinsically self-controlled in B cells.

## IgE CSR Regulation

**Figure 1:**
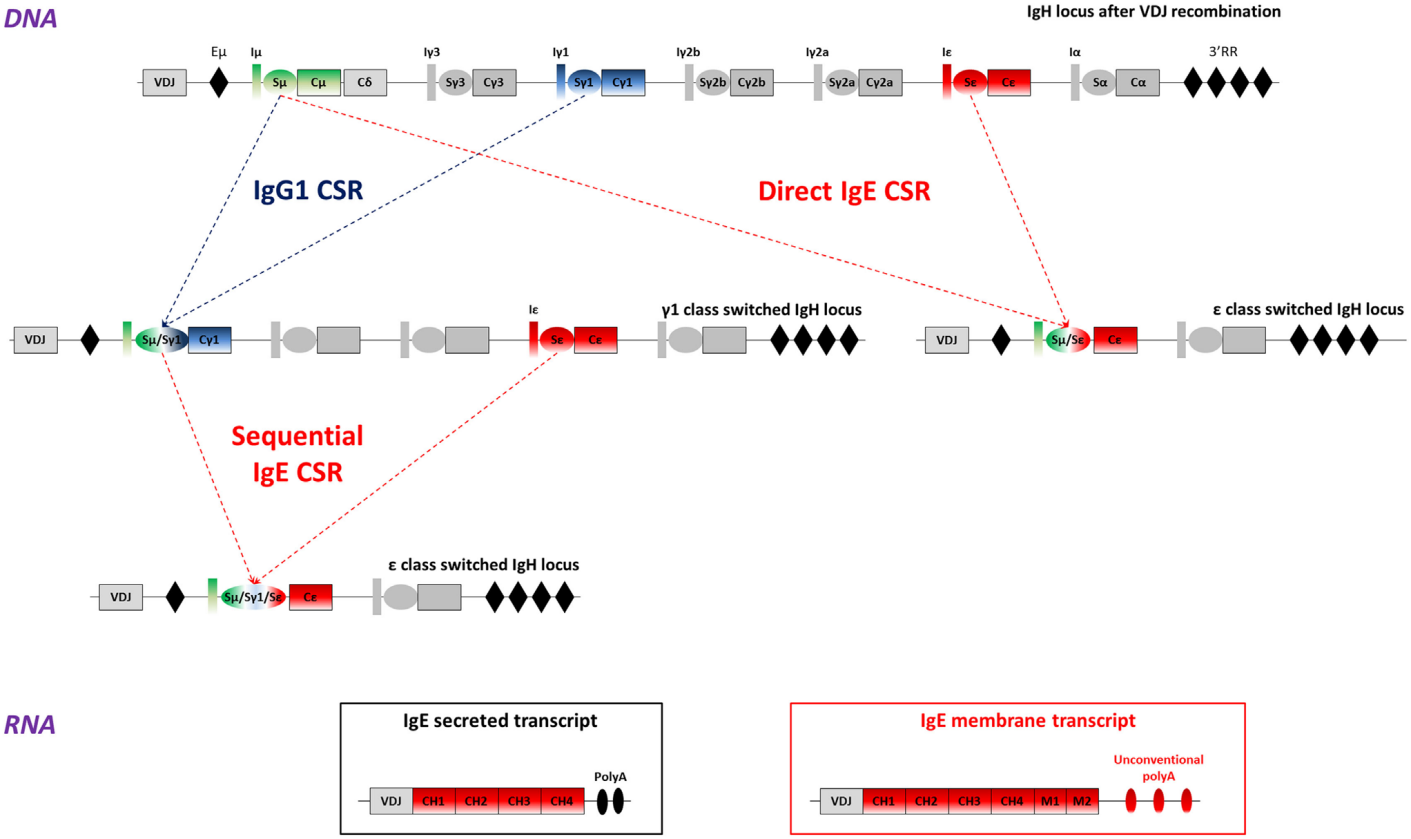
IgE class switch recombination, secreted, and membrane IgE (mIgE) transcripts. Top: mouse WT IgH locus is represented after VDJ recombination (not to scale). The recombined VDJ gene and constant (C) genes are represented as outlined boxes. I exons (I) and switch (S) regions preceding each constant gene (excepted Cδ) are represented as boxes and ovals, respectively. Black diamonds portray important regulatory elements: the intronic enhancer μ (Eμ), and the super-enhancer 3′ regulatory region (3′RR). *Upper-middle*: after B-cell activation, activation-induced deaminase (AID) is expressed and exerts its mutagenic activity on the VDJ gene, allowing production of antibodies with increased affinity for the Ag. AID is also targeted to S regions to initiate DNA double strand breaks, thereby inducing class switch recombination (CSR) between two S regions, here Sμ and Sγ1, or Sμ and Sε. This process allows the production of high-affinity IgG_1_ antibodies, or low-affinity IgE. *Lower-middle*: cells can undergo more rounds of cell cycle or re-express AID after re-exposure to antigen and undergo a sequential CSR event, from γ1 to ε here, to produce high-affinity IgE antibodies. *Bottom*: IgE class-switched cells produce two main transcripts: mIgE transcripts including the four constant exons (CH1 to CH4), the two membrane (M1 and M2) anchor domains and unconventional polyadenylation (polyA) signals and/or secreted IgE transcripts, containing the constant genes and two conventional polyA signals. Alternate IgE transcripts have been described and could contribute to IgE response regulation.

Class switch recombination occurs between switch (S) donor and acceptor regions (Figure 1). S regions are preceded with non-translatable I exons and I promoters, regulated both by the cytokine and cellular environment and in a B cell intrinsic manner. The IgH super-enhancer 3′ regulatory region (3′RR), a hub for multiple factors important for B-cell identity and/or activation is a major regulator of CSR (24, 25). Following B cell activation, germinal transcription of I-S regions is induced, recruiting AID, which initiates DNA double strand breaks under the control of the 3′RR [for review (26)]. Synapsis between broken S regions is also 3′RR dependent (27).

I promoters are crucial CSR regulators for each isotype by carrying sites for specific transcription factors. The Iε promoter binds E2A, AP1, C/EBP, STAT6, PU.1, Pax5, and NF-κB (28, 29). Classically, Iε-Sε transcription before IgE CSR is induced by phosphorylation and dimerization of STAT6, which is stabilized by SWAP-70 (30). While IL4 induces STAT6 and NFIL3/E4BP4, CD40 ligation synergistically activates NF-κB (31). In contrast, several negative regulators inhibit Sε transcription and CSR. Bcl6, strongly expressed in GC, competes with STAT6 for the same binding sites, then restricting IgE CSR. Id2 sequesters E2A and Pax5 and inhibits Iε transcription (32) [for review (33)]. TGF-β, IFNγ, and IL10 likely contribute to these negative regulations, and in human by concomitant binding of HoxC4 and Oct-1 to the Iε promoter (34).

Sε structure itself contributes to IgE CSR regulation, as the shortest and least repetitive S region in both mouse and human (2 kb only, whereas Sγ1 is around 10 kb) (35). Consistently, either Sε replacement by Sμ or insertion of Cε downstream of Sγ1 enhances IgE production (36, 37). Epigenetic changes also affect Sε: H3 acetylation and K4 tri-methylation (38) and IL4-dependent demethylation (39). Immature B cells, with potentially different epigenetic marks, thus preferentially switch to IgE rather than IgG_1_ (40).

IgE CSR can be direct (Sμ to Sε) or sequential (Sμ to Sγ1 first and then to Sε) with an IgG_1_ intermediate (41) (Figure 1). Direct CSR produces low-affinity IgE that competes with high-affinity IgE generated by sequential CSR (42). A probabilistic model suggests that low activation induces IgE CSR while high level favors IgG_1_ CSR with eventual secondary CSR to IgE (43). This could impact *in vivo* IgE responses which can be either inside or outside the GC, respectively, providing stronger or weaker co-receptor signaling. Human IgE^+^ cells are also produced by either direct or sequential CSR (44). Taken together, these features limit Sε accessibility during GC reactions, ensuring a first control for IgE production in cells rather biased toward preferential IgG_1_ CSR (45).

## Multiple IgE Membrane and Secreted IgE Transcripts

After IgE CSR, B-lymphocytes express mIgE heavy chain transcripts, containing four constant (CH) and two membrane exons (M1 and M2), while PCs produce secreted transcripts lacking membrane exons (Figure 1). Alternatively spliced human IgE transcripts are found both for secreted and membrane forms, and can notably include a supplemental sequence of 156 nucleotides using an acceptor splicing site upstream of the M1 exon (46–50). Human mIgE BCR variants thus feature short _mS_IgE and long _mL_IgE, expressed after B cell activation (51). Alternately spliced IgE transcripts were also found in the mouse (52).

Noticeably and contrary to other Ig genes, membrane-type IgE transcripts lack a canonical AATAAA polyadenylation (polyA) site downstream of membrane exons. Maturation of these mRNAs then relies on suboptimal variant polyA sites likely lowers mIgE expression. The resulting ratio of secreted-type/membrane-type ε mRNA is indeed higher than for IgG in stimulated B cells, but partially normalized upon insertion of a classical polyA site downstream the IgE membrane exons *in vitro* (53). However, the insertion of a classical polyA sequence downstream the IgE gene does not really increase IgE level *in vivo* (54). Unconventional polyA sites are found in many sequenced organisms, notably 26 primate and 12 non-primate species (55).

Altogether, this alternative and non-canonical maturation of IgE transcripts could dampen IgE production, notably with low mIgE expression conferring specific properties to mIgE^+^ cells. In other models, low BCR expression was shown to boost tonic BCR signaling and plasma cell differentiation (56, 57).

## IgE^+^ Lymphocytes are Prone to Differentiation into Short-Lived PCs

Following Ag activation, some cells differentiate into memory B cells (58), while others evolve into short- or long-lived PCs, then ensuring short-or long-term infusion of specific Ig in body fluids.

Studying IgE^+^ cell fate *in vivo* in WT mice suggested that these cells transiently appeared outside GCs and then rapidly yielded PCs (59). This differentiation was also observed in a transgenic model, where IgE^+^ cells become GFP^+^, with IgE^+^ cells overexpressing Blimp-1 and prompt to become short-lived PCs (60). This effect is also observed *in vitro*, using feeder cells and cytokines for B cell stimulation, exclusively generating IgM^+^ and IgG_1_^+^ B cells, while IgE^+^ cells again rapidly become PCs (61). Other *in vitro* B cell stimulation protocols also suggest PC predisposition for IgE^+^ cells expressing a WT or an exogenous mIgE (62, 63).

We also found exacerbated PC differentiation *in vivo* within the LATY136F mouse model, with a ratio of IgE^+^ plasmablasts vs. B-lymphocytes 10- to 100-fold higher than for IgM and IgG_1_ (64). Transfer experiments into immunodeficient mice showed a rapid collapse of these IgE^+^ cells, both lymphocytes, and PCs, arguing for a short-lived fate.

Normal B cell differentiation and activation are known to involve mobility, notably into GC. *In vitro* migration assays, by contrast, revealed that cells expressing mIgE poorly migrate toward chemokines (64). This likely compromises their entry into optimal zones of long-term survival as memory B cells or even as long-lived PCs in physiological conditions.

**Figure 2:**
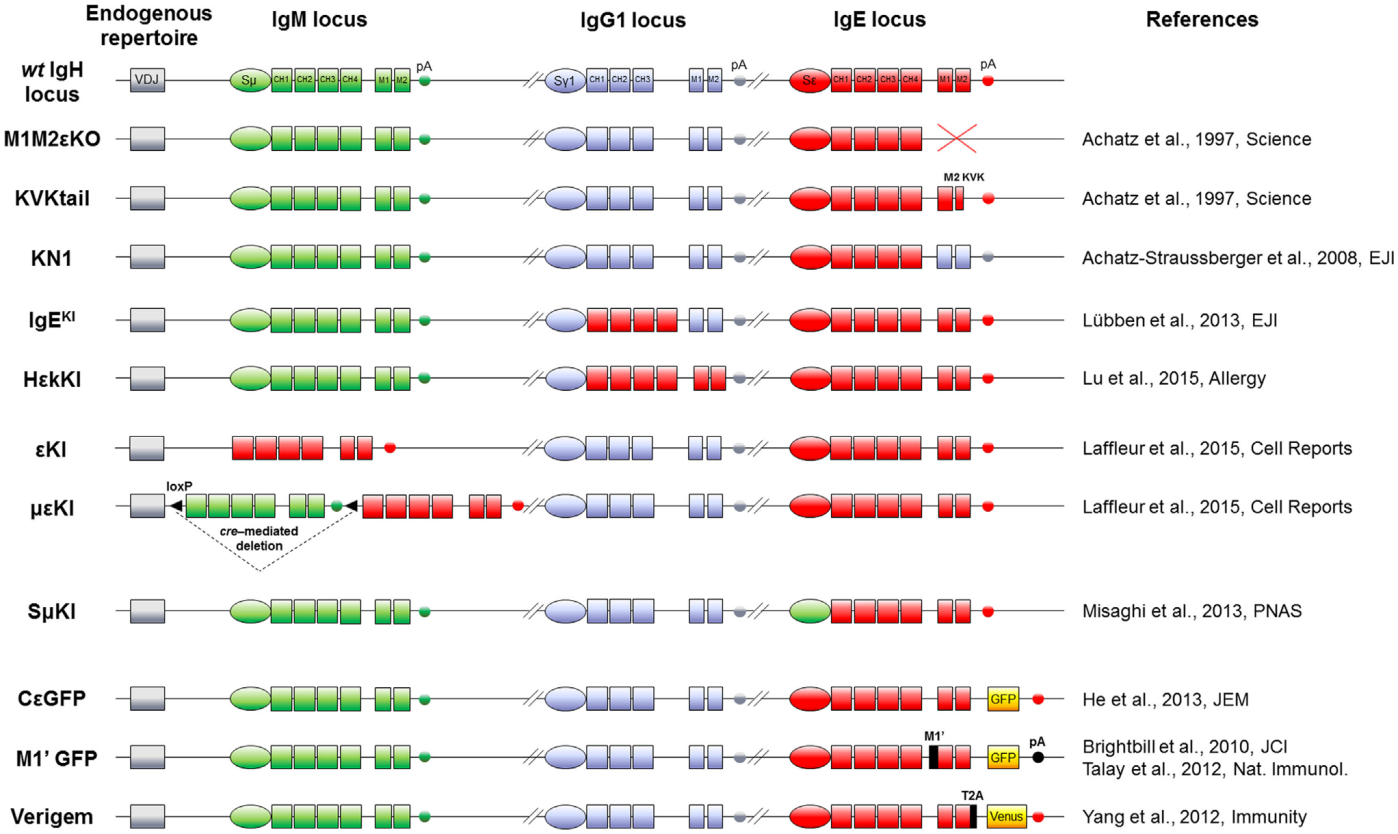
IgH mouse models to study IgE responses *in vivo*. Top: mouse WT IgH locus is represented after VDJ recombination (not to scale). IgM (green), IgG_1_ (blue), and IgE (red) loci including their switch regions (S), constant exons (CH), membrane genes (M), and membrane polyadenylation sites (pA) are shown. *Middle*: knock-out and knock-in alleles are represented, using the color code (IgM elements in green, IgG_1_ in blue and IgE in red) for modifications of switch regions, exons and polyadenylation sites. *Bottom*: the three last models feature insertion of fluorescent reporter genes (yellow boxes) and exogenous sequences (black). Name and references of each mouse model are specified on sides.

That IgE plasmablasts have an intrinsic lower chance to migrate to long-lived PC niches was indeed demonstrated *in vivo*, using chimeric BCR IgE/IgG_1_ [Cγ_1_ membrane exons knock-in (KI) into Cε, Figure 2] (65). However, long-term allergy obviously involves the continuous infusion of specific IgE into the blood by long-lived PCs, which may then constitute the only cell compartment surviving after IgE CSR. Such PCs can indeed be demonstrated *in vivo* in spleen and bone marrow (66). Also in a model of forced IgE CSR, mIgE-expressing cells disappeared within a few days following induction, while IgE secretion persisted for months (64). In human, bone marrow transplantation can induce the transfer of allergen-specific IgE production, likely by transferring long-lived PCs (67). Of note, IgE serum half-life is less than 2 days, ensuring their rapid disappearance if secretion ceases. Although this half-life is higher for cell-bound IgE carried by FcεRI-expressing cells (68), only the permanent production of IgE by persisting long-lived IgE PCs can account for lifelong allergic conditions. Beside immediate allergy related to persistent IgE secretion but with some delay after an antigenic boost, IgG memory B cells, can also generate new IgE-secreting cells after sequential CSR.

It thus appears that the actors of long-term IgE responses are mostly PCs. Despite their scarcity, their persistent production of a highly pro-inflammatory Ig class endows them with a major role in both physiology and pathology. Since IgE CSR is very efficient *in vitro* and since many reports showed that IgE-switched cells more efficiently differentiate into PCs than into persisting IgE^+^ lymphocytes, it remains to be understood why IgE PCs are so rare *in vivo* and IgE production so low (about 1,000-fold below that of specific IgG). Convergent data now show that the rate-limiting step of IgE PC generation is a specifically fragile and short-lived status of mIgE^+^ lymphocytes. Poorly moving and briefly surviving, these mandatory precursors (69) constitute a bottleneck in IgE PC generation.

## mIgE Expression Strongly Impacts Lymphocyte Cell Fate

**Figure 3:**
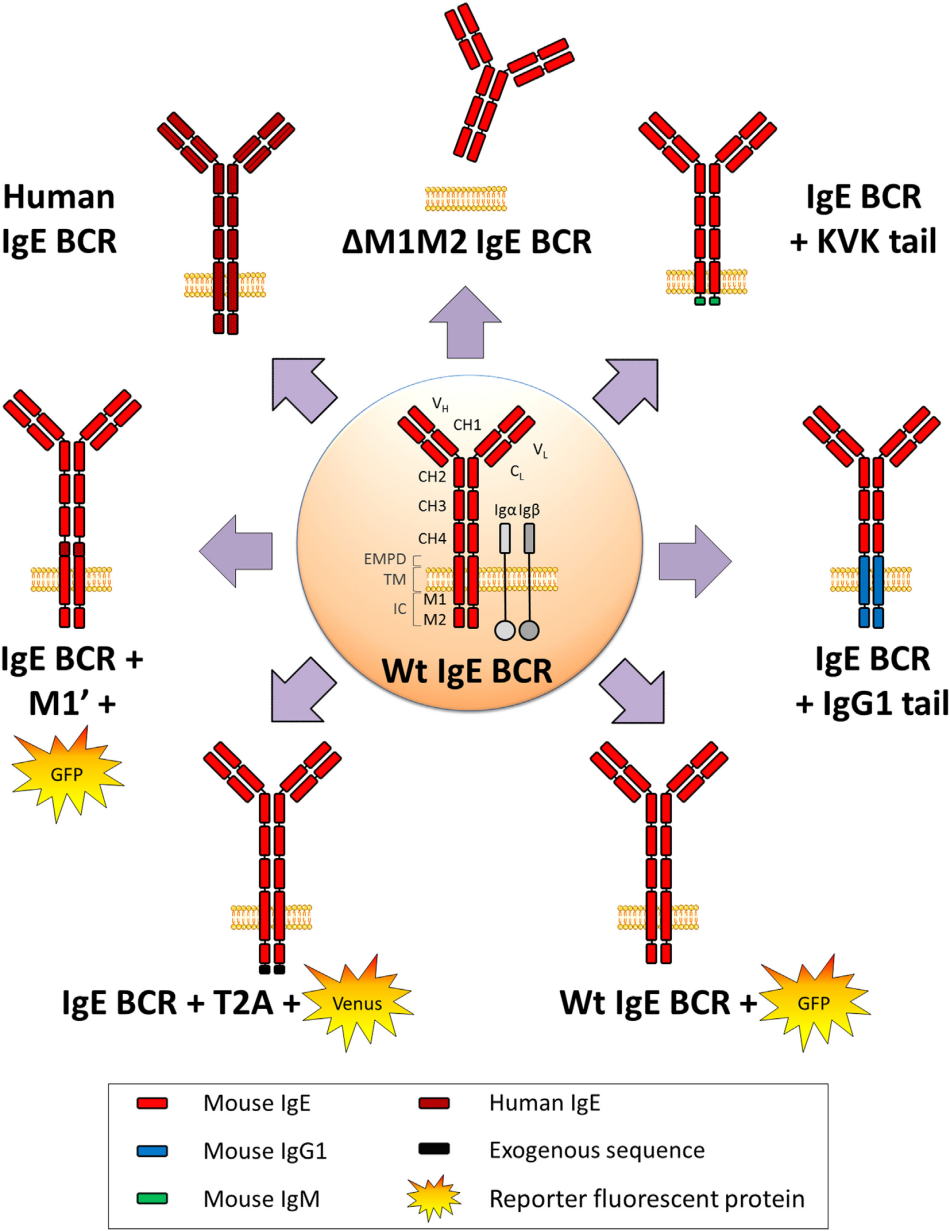
WT and variant IgE B cell receptor (BCR) expressed in mouse models. Mouse WT IgE BCR structure is schematized in the center. Heavy and light chain variable (VH and VL) domains form the antigen-binding site, constant domains (CH), transmembrane (TM), and intracellular domains (IC) are shown, and anchored in the lipid membrane. IgE BCR includes an extra-membrane proximal domain (EMPD). Co-signaling molecules Igα and Igβ are represented in gray. Variant and chimeric IgE BCR resulting from the different knock-in and knock-out alleles (described in Figure 2) are represented, and reporter fluorescent proteins are illustrated.

In physiological conditions, mIgE^+^ lymphocytes are barely detectable *in vivo* either in mouse or human. They are, however, mandatory for building IgE responses, knock-out (KO) of membrane exons (Figures 2 and 3) decreasing total serum IgE by 95%, and preventing the production of specific IgE after immunization (69). Replacement of the IgE intracellular tail with a KVK tail (similar to IgM BCR) (Figures 2 and 3) also decreased IgE production, showing that this intracellular tail positively contributed to IgE secretion (69). That antibody targeting the mIgE extracellular portion decrease IgE production *in vitro* as well as *in vivo*, further underlines the importance of mIgE^+^ cells as IgE PC precursors. A first study showed *in vitro* induction of apoptosis of mIgE^+^ cells and inhibition of specific IgE responses in allergic mice, using an anti-EMPD (extracellular membrane-proximal domain) monoclonal antibody (MAb) (70). Anti-human extra-membrane proximal domain (EMPD) antibodies proved efficient *in vivo*, targeting humanized mouse (Figure 2), or human PBMC, by promoting apoptosis, decreasing IgE PC number, and lowering IgE production in models of asthma induction and helminth infection (71). Soluble IgE neutralization and decrease of IgE^+^ cells was also obtained using a single-chain antibody against total IgE (72). Another antibody targeting total IgE and including a FcγRIIb binding Fc region is 40-fold more efficient than the classical omalizumab MAb anti-IgE in HuSCID mice, diminishing PCs and IgE production, likely due to co-engagement of mIgE and the inhibitory receptor FcγRIIb at the lymphocyte surface (73). It remains to be explored whether the IgE BCR is only expressed on B-lymphocytes, as for IgG, or also on some bone marrow long-lived PCs, as for some IgA and IgM-PCs (74, 75). Altogether, mIgE^+^ cells are a mandatory step of IgE responses, but since they poorly show up *in vivo*, their study is challenging and has needed specific biochemical and cellular *in vitro* and *in vivo* strategies.

Phage display helped identify two mIgE intra-cytosolic binding proteins: HPK1 binds mIgE and other isotype tails (76) while HAX1 only binds IgE (77). IgE production, however, remained unaffected in corresponding KO models (78–80).

As for transfected IgE BCRs expressed *in vitro* in cell lines, both human mIgE forms expressed into the immature WEHI cell line, associated with mouse signaling co-receptors Igα/Igβ, and inhibited cell growth after _mS_IgE BCR cross-linking (51). Chimeric receptors (missing CH1 to CH3 exons) expressed in A20 mature B cell line induce caspase-dependent apoptosis upon cross-linking when lacking the EMPD domain (81). Short chimeric constructions expressed into Ramos human mature cells showed Grb2 adaptor recruitment to intra-cytoplasmic mIgG or mIgE tails, enhancing BCR signals and proliferation (82). Membrane IgE expression alone induced some apoptosis of the mature Daudi cell line, which was increased by mIgE cross-linking and reversed by caspase inhibitors (71).

Primary cell studies need tricky sensitive protocols to specifically identify true mIgE^+^ cells and not passive IgE binding to Fcε receptors. Several protocols limit false positivity: cells can be intracellularly stained after trypsin treatment, to remove CD23-bound IgE (40), or after saturation of surface IgE with non-fluorescent antibodies (60). An acidic wash can also efficiently remove CD23-bound IgE and then allow for direct mIgE staining (59).

*In vitro*, IL21 induces apoptosis of IgE^+^-stimulated B cells, but not IgG_1_^+^ (83). High concentration of IgE^+^ cells also promotes caspase-dependent apoptosis (84). Using a more sophisticated *in vitro* system, “GC like” structures can be created with IgM/IgD and IgG, but not IgE, “memory” B cells, suggesting that mIgE^+^ cells die (61).

We observed spontaneous apoptosis induced by the human mIgE expression, both in heterologous (mouse mature B cell line) and homologous (human mature B cell line) transfectants. Consistently, we observed increased apoptosis of mIgE^+^ lymphocytes among primary B cells stimulated *in vitro* toward CSR and deprived of cytokines. This apoptosis implicated the mitochondrial and caspase pathways and was partially reversed using cyclosporine A, caspase inhibitors, or bcl2 overexpression. In these cells, survival was affected by multiple means: mobility was drastically reduced while the actin cytoskeleton was reorganized and cells took circular shapes, HAX1 was delocalized from mitochondria, IgE BCR was spontaneously incorporated into lipid rafts and endocytosed, transcriptional program controlling cell death, metabolism, signaling, and mobility was affected (64). Another study confirmed spontaneous apoptosis *in vitro*, upon expression of an exogenous BCR into stimulated primary B cells, and confirmed that the IgE BCR induces cell death actively, and not due to a signaling defect (63).

All these data are informative about the B-cell intrinsic impact of mIgE expression but do not integrate the *in vivo* complexity of IgE responses. Beyond these intrinsic aspects, extracellular factors such as cell–cell interactions, Ag presentation by B-cells to T-cells and various soluble factors could strongly influence IgE^+^ B cell fate. Methods and mouse models have been developed by different labs and help further understand IgE responses.

## WT and Transgenic Mouse Models to Investigate IgE Responses *In Vivo*

One first model involved a transgene encoding secreted IgE, thus increasing serum IgE level, but without providing any clue about mIgE^+^ cells (85). KI insertion of Cε into the Cγ_1_ locus (Figures 2 and 3) also increased IgE production without generating mIgE^+^ B cells *in vivo*, whereas these cells appeared *in vitro* (37). IgG_1_ membrane exons (M1–M2) KI downstream of Cε (Figures 2 and 3) also increased IgE basal levels and secondary IgE responses, suggesting that the transmembrane (TM) and intracellular IgE tail contribute to IgE homeostasis *in vivo* (65). De Lafaille and colleagues developed a hyper-IgE model revealing several aspects of IgE responses: these mice carry a transgenic anti-OVA TCR and a KI anti-HA BCR, inducing strong IgE response after immunization with OVA-HA peptide and revealing the role of T_REGS_ in controlling IgE production (22). In this model and in helminth (*Nippostrongylus brasiliensis*)-infected WT mice, IgE^+^ PCs were localized outside GC, whereas IgE CSR occurred in GC IgG_1_^+^ cells, i.e., through sequential CSR (Figure 1). Intracellularly stained IgE^+^ cells have specific characteristics: they are large, express PC genes but not CXCR5, which is implicated into GC localization (59). Ig affinity was studied after OVA-PEP1 immunization (a variant of OVA-HA), and showed rapid specific IgG_1_ but delayed specific IgE production, suggesting sequential CSR from IgM to IgG_1_ and then to IgE, where SHM occurs in parallel to IgG_1_ CSR (59). Consistently, IgG_1_-deficient mice show a defect in specific IgE production, whereas total IgE level is unaffected. This suggests that sequential IgE CSR generates high-affinity (potentially pathogenic) IgE, whereas direct IgE CSR generates low-affinity IgE (rather dampening allergy by saturating FcεRI receptors) (42). We can note that a “natural” IgE production by B2 cells, independently of MHCII, could eventually provide some of the protective low-affinity IgE antibodies (86), as well as IgE produced by immature B cells (40). The specific role of IgG_1_ memory cells into IgE memory has been confirmed using a high-throughput sequencing approach and adoptive transfer, suggesting the absence of mIgE^+^ memory B-lymphocytes (87).

Three fluorescent reporter mouse models have been generated to study the rare IgE^+^ cells *in vivo*. The first one (M1’ GFP) includes a human EMPD IgE domain knocked into the mouse Cε (Figure 2) and an IRES-GFP cassette downstream of membrane exons. Upon *N. brasiliensis* infection, IgE^+^ cells were then detected into GCs, but without strong PC differentiation (54). These conclusions could be biased by this chimeric BCR, the use of a classical polyA for membrane exons, and the fact that this IRES does not tag the IgE protein but IgE transcripts (with some GFP^+^ cells expressing functional IgG_1_^+^ on one hand and germline Cε transcripts on the other). A second model used the peptide 2A system to really tag IgE^+^ cells, coupling translation of mIgE and the fluorescent protein “Venus” (Figure 2), so that IgE^+^ cells are also Venus^+^. This model showed IgE^+^ cells into GC and confirmed their PC bias. These PCs were mostly short-lived but their number can be enhanced by Bcl2 overexpression, they express mIgE, opposite to IgG PCs (60). Another IRES-GFP cassette has been inserted into Cε, conserving the natural architecture of mIgE and the unconventional polyA sites (Figure 2). This showed that along *N. brasiliensis* infection, IgE^+^ cells generated into GC were less mobile than IgG_1_ and restricted to the dark zone, limiting their contribution to memory. This also showed more *in vivo* apoptosis of IgE^+^ than IgG_1_^+^ lymphocytes (88).

All these models converge toward the conclusion that the IgE response highly differs from other isotypes, by generating few class-switched lymphocytes, which are short-lived and might then face the cell fate decision of differentiating into PCs or dying through apoptosis. Since there are good indications that mIgE by itself elicits signals that cut B-cell mobility, shorten survival, and/or induce apoptosis (64), the issue of BCR expression in long-lived PCs downstream of the B-lymphocyte stage is of interest. For IgM^+^ and IgA^+^ PCs expressing a functional BCR, cross-linking this receptor with high doses of anti-BCR antibodies decreases PC survival (74). Regarding the mIgE which by itself shortens the half-life of B-lymphocytes (somehow mimicking high-dose cross-linking of other BCR classes), whether it can also be detrimental to PC survival remains an open issue.

Two mouse models uncoupled mIgE expression to normal CSR and microenvironment signals. Cε was inserted into Sμ (εKI, Figure 2) to force mIgE BCR expression in place of mIgM, as previously done successfully with mIgA (89). A strong blockade of early B cell development hereby occurred, suggesting a defect in ε − pre-BCR signaling or a pro-apoptotic signal. Another model followed the same strategy, but now including a floxed human Cμ upstream of Cε (μεKI, Figure 2), human IgM expression then initially supporting B cell development (90). Upon tamoxifen-inducible cre-deletion, these mice secreted IgE and transiently showed IgE^+^ B-lymphocytes. IgE levels peaked around 1 week after induction and while IgE^+^ B-lymphocytes rapidly vanished from lymphoid tissues after a few days independently of any BCR cross-linking, secreted IgE levels persisted for months, indicating that long-lived IgE^+^ PCs survived (64). Another model replaces Cγ1 with human Cε but conserving Sγ1 and its polyA site (Figure 2). Human IgE level was then higher than mouse IgE, but much lower than IgG_1_. Very infrequent IgE^+^ hybridomas could be obtained after immunization, suggesting again an underrepresentation of mIgE^+^ cells (91).

While expression of human IgE in a mouse context could participate to this phenotype, this was consistent with the observation of increased apoptosis in IgE class-switched primary B-cells. A recent study also confirmed spontaneous apoptosis of IgE^+^ lymphocytes, induced by mIgE expression in a BLNK-dependent manner, while the same cells were also simultaneously induced to leave the B-cell stage and readily become PCs, with a PC bias attributed to the CD19 and BLNK signaling pathways. This was dependent from mIgE extracellular domains (63). FAS-dependent apoptosis (92) might also dampen IgE responses since FAS inactivation yields expanded IgE PCs, and IgG and IgE autoantibodies (18).

Finally, a recent study showed that expression of mIgE promotes PC differentiation rather than survival of mIgE^+^ lymphocytes even in the absence of Ag, due to constitutive activation of Syk, CD19, BLNK, Btk, and IRF4 signaling pathways. This model did not demonstrate apoptosis but a proliferative defect and showed an exaggerated GC response for IgE^+^ B cells after disruption of BCR signaling. IgE^+^ B cells also poorly presented Ag *in vivo*, which could explain poor participation to immune responses (62).

Altogether, IgE^+^ B cells are very scarce *in vivo*, they can be generated for a short-term window but quickly vanish, apparently only surviving as PCs.

## Discussion

### IgE and Their Receptors

IgE responses are also controlled by IgE receptors, several of which exist under soluble as well as membrane-anchored isoforms. Surface FcεRI expression in mast cells is stabilized by soluble IgE (93) and participates to the clearance of soluble IgE by dendritic cells and monocytes (94). Membrane FcεRI, expressed by a basophilic leukemia cell line, also bound mIgE expressed by lymphocytes (95). The soluble form of FcεRII/CD23 also binds mIgE (96), and interestingly, FcεRII KO increases IgE responses (97). FcγRIV can bind IgE-Ag immune complexes and activate macrophages in mouse (98). Other receptors like soluble galectin 3 and 9 also bind IgE, respectively, with low [for review (99)] or high affinity (100), and might also bind mIgE. We can speculate that this hub of IgE receptors, expressed and secreted by different cell populations or by mIgE^+^ lymphocytes themselves, and IgE/Ag immune complexes could cross-link mIgE, re-enforcing the intrinsic control mIgE^+^ lymphocyte homeostasis.

### IgE Memory

After primary immunization in the mouse, multiple layers of memory B cells are generated, including IgM^+^ and IgG^+^. This ensures secondary responses with rapid PC differentiation of IgG^+^ memory cells and re-entry into GC center of IgM^+^ memory cells (101). Long-lived PCs establish soluble Ig memory for long term. Class-switched BCRs strongly influence B cell fate, globally inducing PC differentiation (56, 89, 102) and modulating the memory B cell pool. As discussed above, there are contradictory data about IgE memory. Some studies suggested that mIgE^+^ memory B cells persisted after IgE responses (54), but convergent reports rather documented that these cells end into short-lived PC and undergo multiple counter-selection processes including apoptosis (36, 37, 60, 64, 87, 88, 91). These contradictory conclusions might result from technical pitfalls, like false mIgE staining, use of leaky IRES, disruption of IgE BCR natural architecture, an introduction of exogenous sequences, heterologous systems, genetic background, etc. Kinetic and spatial issues are also crucial as IgE responses are transient and restricted to specific compartments, which is difficult to investigate *in vivo*. Differences between mouse and human are also possible.

Numerous indirect pieces of evidence strongly suggest the absence of mIgE^+^ cells in the memory lymphocyte compartment; thanks to sequential CSR to IgE after an antigenic boost, some IgE memory could in fact indirectly be created through *de novo* CSR of classical IgG_1_^+^ memory lymphocytes then ending with the production of high-affinity IgE. This mechanism could ensure that high-affinity IgE antibodies are not generated as a first defense layer but only in a second stage when Ag reappears in an appropriate cytokine context and that these responses are essentially transient.

**Figure 4:**
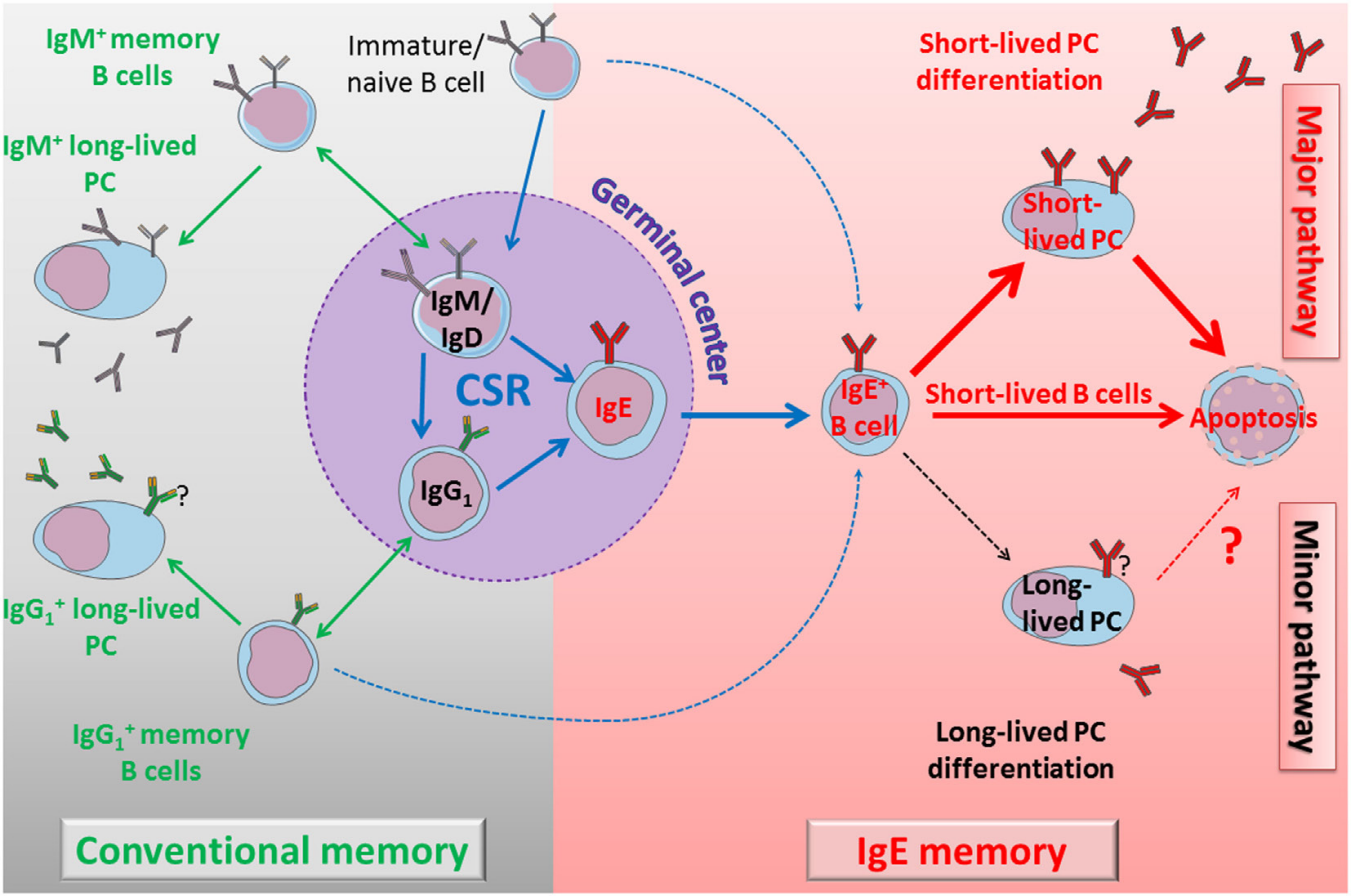
Self-controlled IgE production model. *Left part*: immature cells differentiate into naïve B cells and could undergo direct class switch recombination (CSR) to IgE or enter into the GC. During the GC reaction, cells can switch directly to IgG1 or IgE, or eventually undergo a sequential switching with an IgG1 intermediate and an extra-follicular CSR to IgE. IgM^+^ and IgG1^+^ lymphocytes generate classical humoral memories, with memory B cells and long-lived plasma cells (PCs). These memory B cells can undergo more rounds of GC reaction to increase their antigen (Ag) affinity and to switch to IgG1 or IgE. Direct or sequential CSR produces membrane IgE^+^ lymphocytes with different Ag affinity, and IgE B cell receptor (BCR) seems to induce a common, short-lived fate. *Right part*: IgE^+^ lymphocytes have a bias in short-lived PC differentiation. These short-lived PCs strongly express IgE BCR and undergo massive apoptosis. Membrane IgE^+^ lymphocytes are also counter-selected by various mechanisms including poor mobility, low membrane IgE expression, and spontaneous apoptosis. Long-lived PCs are very elusive but produce IgE antibodies for a long term, potentially including pathological IgE. A better characterization of these cells and their targeting to eliminate them could lead to new treatments to re-initialize IgE memory and deplete pathological IgE production.

Membrane IgE expression strongly affects lymphocyte phenotype and we suggest that evolution selected multiple mechanisms to restrict IgE responses. IgE tail itself perturbs BCR signaling, eventually by sequestrating anti-apoptotic protein (HAX1), recruiting adaptor or signaling proteins (Grb2, Syk), or through still unknown interactions. TM and extra-membrane domains notably contribute to specific interactions with Ig-α, CD19 and probably other molecules. IgE is highly flexible (103, 104) and could spontaneously adopt a closed conformation, even when expressed as a BCR, and be spontaneously cross-linked; this effect could be enhanced by interactions with IgE receptors. Cε domains seem to be implicated in multiple signaling pathways and functions, ensuring both efficient PC differentiation and a selective disadvantage for mIgE^+^ cells, with reduced proliferation, Ag presentation, and mobility, also affected with constitutive BCR clustering into lipid rafts and endocytosis lowering surface expression, globally promoting poor survival. These effects are now well documented in lymphocytes and might also impact mIgE^+^ PCs (Figure 4).

#### Note Added in Proof

A new mouse model has been generated to force membrane IgE expression from early B cell development (105), similarly to εKI mice (Figure 2), but using a different strategy and a mouse Cε gene. These animals have also a strong blockade at the pro-B cell stage, again showing that Cε gene cannot support B cell development. Interestingly, the authors demonstrate that the strong (secreted vs. membrane) ratio of IgE accounts for this phenotype, which could be partially rescued by inserting an mIgE. Finally, they show that increasing BCR signaling by PTEN deletion increases mIgE cell number and IgE responses.

## Conclusion

IgE production is tightly regulated, from CSR to PC differentiation, including IgE half-life and survival of mIgE^+^ memory B cells. It is interesting to note that upon all different KI strategies deployed to investigate IgE^+^ cells *in vivo* (Figure 2), these cells are generated at a very low frequency, only during short intervals, and do not yield mIgE^+^ memory B cells. IgE CSR is initially controlled by the cytokine microenvironment, the anomalies of which can boost IgE production in allergic individuals (106). The mechanistic of Sε accessibility likely contributes to restricting IgE CSR in mature B cells, as indicated by Sε replacements (36). Unconventional polyA sites could also contribute to IgE regulation (53). Several chimeric IgE BCR models were generated to dissect domain functions (Figures 2 and 3), but it seems that distinct protein parts contribute to disadvantage mIgE^+^ cells, resulting in overrepresentation of short-lived PCs and almost no accumulation of mIgE^+^ lymphocytes. The protracted expression of the IgE BCR might also provide counter-selective signals in plasmablasts and contribute to their short-lived fate. These multiple mechanisms induced by mIgE expression even in the absence of Ag could be enhanced *in vivo* by mIgE cross-linking.

Membrane IgE expression *per se* is, thus, a powerful gatekeeper of IgE production, both ensuring and restraining a punctual IgE response while minimizing IgE memory. These elements clearly need to be taken into account when dealing with therapy of IgE-mediated disorders. Acting on cytokines and microenvironment of the immune response is important at the initiation of IgE responses and for preventing sequential IgE CSR of memory B-lymphocytes (notably IgG1). Acting on the true memory cells responsible for the long-term infusion of IgE should by contrast rather focus onto IgE PCs.

## Author Contributions

BL and MC wrote the manuscript, OD created the figures and participated to the redaction, and ZD helped for references and redaction.

## Conflict of Interest Statement

The authors declare that the research was conducted in the absence of any commercial or financial relationships that could be construed as a potential conflict of interest.
